# Spatio-temporal patterns of tigers in response to prey species and anthropogenic activities

**DOI:** 10.1098/rspb.2024.1939

**Published:** 2025-01-29

**Authors:** Hari Prasad Sharma, Bishnu Prasad Bhattarai, Sandeep Regmi, Pei-Jen Lee Shaner, Shivish Bhandari, Amrit Nepali, Bishnu Aryal, Krishna Tamang, Sabin KC, Ajay Karki, Ashok Kumar Ram, Jerrold L. Belant, Hem Bahadur Katuwal

**Affiliations:** ^1^ Central Department of Zoology, Institute of Science and Technology, Tribhuvan University, Kirtipur, Kathmandu, Nepal; ^2^ Nepal Zoological Society, Kirtipur, Kathmandu, Nepal; ^3^ Center for Integrative Conservation, Xishuangbanna Tropical Botanical Garden, Chinese Academy of Sciences, Mengla, Yunnan 666303, People’s Republic of China; ^4^ National Taiwan Normal University, Taipei, Taiwan; ^5^ Department of Biology, Morgan State University, Baltimore, MD 21251, USA; ^6^ Department of National Parks and Wildlife Conservation, Kathmandu, Nepal; ^7^ Department of Fisheries and Wildlife, Michigan State University, East Lansing, MI 48824, USA

**Keywords:** apex predator, conservation, human–wildlife conflicts, *Panthera tigris*, space use, tiger

## Abstract

Understanding factors influencing the spatio-temporal patterns of apex predators is prerequisite for their conservation. We studied space use and diel activity of tigers (*Panthera tigris*) in response to prey availability and anthropogenic activities with trail cameras in Nepal during December 2022–March 2023. We used hierarchical occupancy models to evaluate how prey availability (space use of prey species) and anthropogenic activities (number of humans and livestock) contributed to the tigers’ space use, while accounting for landscape effects on their detection probability. We calculated the diel activity overlap between tigers and each prey species, as well as with humans and livestock. Overall, tigers had relatively high space use (0.540 ± 0.092) and detection probability (0.742 ± 0.073), and were most influenced by space use of wild pig (*Sus scrofa*), gaur (*Bos gaurus*) and number of livestock detections. Tigers exhibited extensive temporal overlap with their prey, but not with humans or livestock. Our study demonstrates that humans and tigers can co-occur in a landscape by altering diel activity and potentially moving cryptically in certain landscapes, provided adequate prey is available. Management actions that ensure adequate prey availability can benefit tiger conservation.

## 1. Introduction

Apex predators provide essential ecosystem functions through their top-down effects [1,2]. The tiger (*Panthera tigris*) is a globally threatened apex predator because of illegal hunting, habitat degradation, land use change and urbanization [3], with an estimated population size of less than 6000 [4], including about 3000 individuals in India [5]. In some areas where tiger populations are recovering, human–tiger conflicts are also increasing owing to limited habitat availability, challenging conservation efforts to increase their population size [6,7]. Understanding how factors such as prey availability and anthropogenic activities shape spatio-temporal patterns of tigers is essential to develop effective conservation strategies.

Tigers mostly prey on large- to medium-sized herbivores such as sambar (*Rusa unicolor*), chital (*Axis axis*), barking deer (*Muntiacus vaginalis*), wild pig (*Sus scrofa*), blue bull (*Boselaphus tragocamelus*) and gaur (*Bos gaurus*) [8–13]. Strong and positive co-occurrence of tigers and their prey underscores the need to consider predator–prey dynamics in tiger conservation [14]. Specifically, the spatial distribution and temporal activity of these prey are key to gain insight into the survival of tiger populations [15,16]. For instance, prey occurrence patterns could inform conservationists where to establish protected areas, corridors and buffer zones, ensuring that tigers have access to areas with adequate prey [17]. Furthermore, as the likelihood of human–tiger conflicts increases with increasing spatio-temporal overlap between humans and tigers [18,19], assessing the risk of tigers encroaching on human settlements in pursuit of prey can help inform conflict mitigation strategies [20–22]. Thus, understanding prey populations are needed not only to maintain viable tiger populations but also to enhance human–tiger coexistence [23,24].

Nepal’s tiger population is increasing [25] but they are mostly confined to protected areas within the transboundary (India–Nepal) Terai Arc Landscape (TAL) [26–28]. At local scales, tiger occurrence largely corresponds with that of their prey [29–32]. However, because the TAL contains a mosaic landscape of forests and agricultural lands, tigers that occur outside protected areas in pursuit of prey are vulnerable to habitat loss from deforestation, development (e.g. road and railway construction) and agricultural land conversion [33]. The Parsa National Park (PNP) westwardly joins with the Chitwan National Park to form important habitats for sustaining tiger populations in eastern TAL [34–36]. Furthermore, PNP contains one of the tiger recovery sites identified under the World Wide Fund for Nature’s Tigers Alive Initiative [37]. In PNP, the tiger population has increased from 18 in 2018 [36] to 41 in 2022 [38], probably reaching the carrying capacity of the area based on prey availability [39].

Tiger occurrence and space use can be influenced by factors including the availability of prey species; anthropogenic factors like settlement, farmlands and linear infrastructure, forest cover and water sources [30,40–42]. Prey availability and forest cover often increases tiger occurrence while anthropogenic factors can negatively impact tiger occurrence [41,42]. Thus, it is necessary to understand how these factors influence tiger occurrence and space use to improve our understanding of tiger ecology to inform management. Such inferences can be gained through methods like occupancy analysis [43,44]. Occupancy studies can provide an alternative method to monitor populations when abundance data are not available or feasible [45]. However, occupancy can be considered space use when there is uncertainty whether species population remains closed throughout the sampling period [44,46].

As the PNP and adjoining forests experience high levels of human activities, the increasing tiger population could lead to increased human–tiger conflicts [47]. Additionally, livestock grazing in these forests can adversely affect ungulate prey availability for tigers [33]. Therefore, the initial success of tiger recovery in this area can only be sustained through effective management of interactions among tigers, their prey, humans and livestock. However, there is currently little information on the spatio-temporal patterns of tigers in this area. We aimed to quantify space use and diel activity of tigers in PNP and adjoining forests and examine how their spatio-temporal patterns of use correspond with prey availability and anthropogenic activities. As tigers can alter their spatio-temporal patterns in response to prey and humans while being sensitive to landscape features [10,19,29,30,32,40,48,49], we predicted that (i) tiger space use and activity will have a positive association with prey and negative association with humans and livestock, and (ii) tiger detection probability will be influenced by landscape features such as distance to water, settlements and roads.

## 2. Material and methods

### (a) Study area

**Figure 1.**
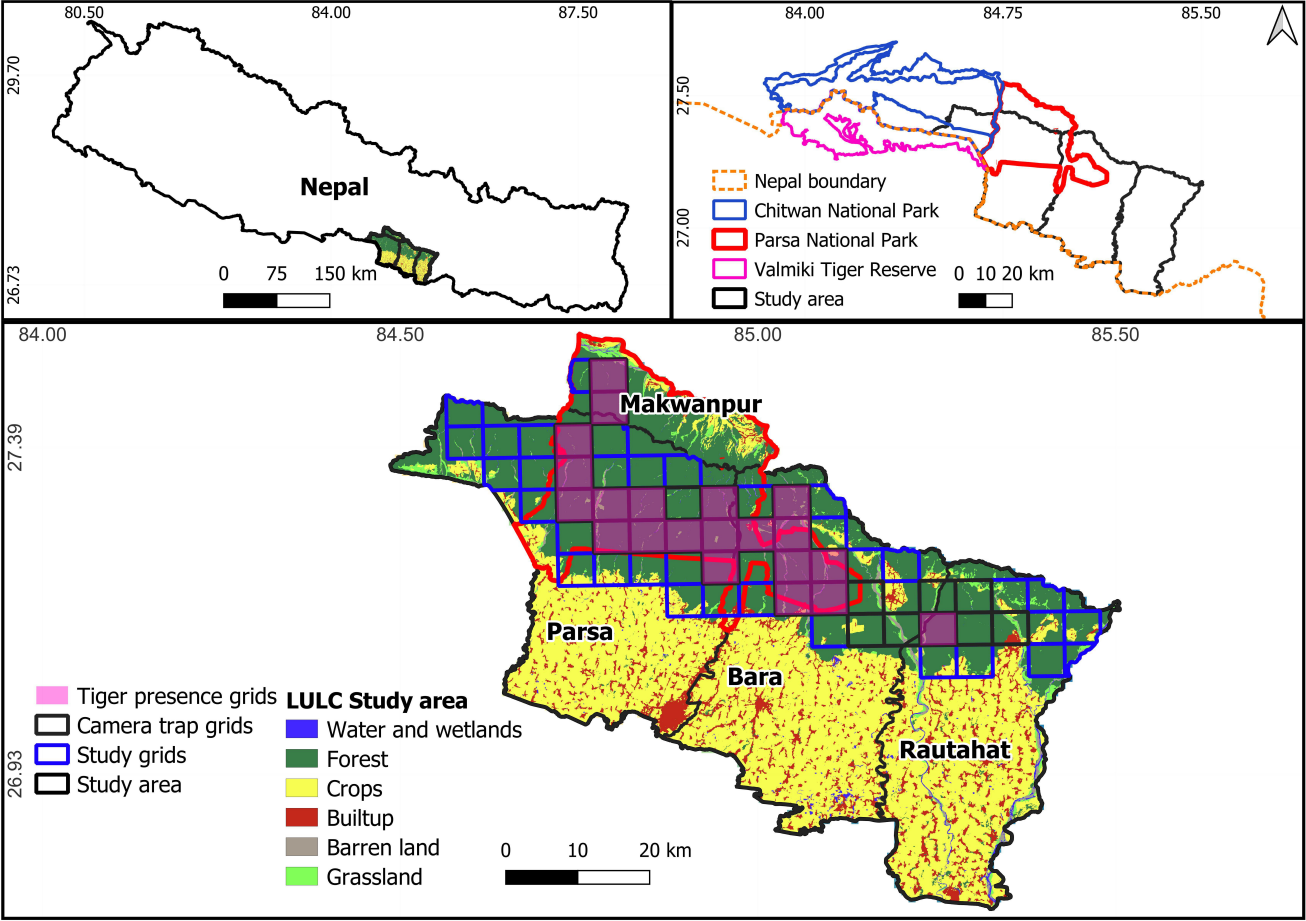
Study area including PNP and adjoining areas, Nepal and its location within the Tiger Conservation Unit Chitwan–Parsa–Valmiki complex, which includes Chitwan National Park and PNP, Nepal and Valmiki Tiger Reserve, India. LULC, land use and land cover change.

This study was conducted in the lowlands of Nepal that includes PNP and adjoining forests (community-managed or government forests in Makawanpur, Parsa, Bara and Rautahat districts of Madhesh Province, Nepal; figure 1), which comprise 3669 km^2^ with elevations of 80−800 m. The area supports over 50 mammalian species contributing to the region’s biodiversity [50–53]. However, anthropogenic activities such as sand extraction, shifting cultivation and domestic cattle grazing are high in this area [54], posing challenges to human–wildlife coexistence.

The study area is within the Tarai and Chure hills of Nepal and includes 90% tropical and subtropical forests with sal (*Shorea robusta),* sisso (*Dalbergia sisoo*), silk cotton tree (*Bombax ceiba*) and khair (*Acacia catechu*) being the dominant tree species and siru (*Imperata cylindrica*) and kans (*Saccharum spontaneum*) dominant grasses. The study area also supports various iconic wildlife species including Asian elephant (*Elephas maximus*), tiger, leopard (*P. pardus*) and sloth bear (*Melursus ursinus*) [53]. Common ungulate species include the blue bull, gaur, chital, sambar*,* barking deer and wild pigs [31] are inhabitants in the study area. These are medium- to large-sized species which make them suitable prey for apex predators such as tigers and leopards [14]. Sambar, gaur and blue bull, in particular, have been suggested to be important prey for tigers [8,12,15,55–57].

### (b) Data collection

We used remote cameras (Stealthcam model STCG45NG, Irving, Texas, USA) to collect the occurrence data of tigers and their prey during December 2022–March 2023. We placed 94 cameras in the study area, each 40−60 cm above ground and positioned toward animal trails, with distance between the nearest cameras of about 1 km. We deployed cameras to correspond with tiger detection zones documented in national tiger surveys conducted in 2013, 2014 and 2016 [58]. We deployed a single camera at each site and programmed the cameras to obtain three images during each detection, with a delay of 30 s between detections. We left the cameras in place for at least 21 days. We identified prey species using a mammal field guide [59].

We included five landscape features from each camera site: (i) distance to the nearest water body; (ii) distance to the nearest major road; (iii) distance to nearest human settlement; (iv) forest cover within a 500 m radius; and (v) canopy cover within 100 m^2^. To estimate the distance to the nearest permanent water bodies (e.g. rivers, streams and lakes), major roads or human settlements from each camera trap, we used either a measuring tape (when distances were < 200 m) or the measure line tool in QGIS (when distances were > 200 m) based on vector files of water bodies, major roads [60] and human settlements [61]. We extracted forest cover within a 500 m radius of each site using 2023 land use data from satellite images (Sentinel-2 10 m resolution land cover) [62]. We measured canopy cover using hemispherical photography (the average canopy cover across four photos each taken at a corner of the 10 × 10 m plot centring around each camera). We recorded human and livestock detections using the same camera images where livestock mainly included cattle or oxen, domestic buffalo and goats.

### (c) Data analysis

#### (i) Occupancy models

We estimated detection probability (*p*) and space use (*psi*) of tigers and their prey using hierarchical occupancy models [63–65]. Herein, we used psi as an estimate of space use rather than true occupancy [46]. We used the term space use as a reflection of the probability of tiger presence within surveyed areas rather than true occupancy, which implies constant presence at a site. Our approach was to capture dynamic patterns in tiger habitat use, which may fluctuate based on prey availability, human presence or other factors. These patterns reflect the probability of tiger occurrence rather than continuous occupancy. Therefore, the space use in this research provides a more accurate description within this context. We conducted adaptive Markov chain Monte Carlo simulation (three chains, 1000 adaptations, a burn-in of 1000 and 10 000 iterations, Rhat value of 1.1 as the threshold of chain convergence) using Just Another Gibbs Sampler [66] in program R [67] to obtain parameter estimates for the models. We used packages ‘coda’, ‘jagsUI’, ‘mcmcOutput’ and ‘rjags’ for the analysis [68–71].

For estimates of prey space use, we included the following variables: total number of humans detected (the sum of the number of humans observed across all photos at a camera), total number of livestock detected (the sum of the number of livestock observed across all photos at a camera), distance to nearest road, distance to nearest settlement, per cent canopy cover, per cent forest cover and the presence of potentially competing species excluding the focal species (e.g. blue bull, chital, gaur, sambar and wild pig for the barking deer model). We assumed a constant detection probability across sites [72,73].

For tigers, we simultaneously modelled space use and detection probability. For each model, we removed variables highly collinear (pairwise correlation > 0.7 (electronic supplementary material, figure S1), and with Variance Inflation Factor (VIF) value > 5 using the ‘vifcor’ function of package ‘usdm’ in R program), retaining the variable considered more ecologically important. For the tiger space use model, we used per cent forest cover, total number of humans detected, total number of livestock detected and space use of three prey species (blue bull, gaur and wild pigs) and remaining prey species were excluded from the model owing to higher correlation. For prey variables, we modelled the ‘psi’ of each prey species and used them as covariates to model tiger space use. We used the number of detections for livestock and occupancy for prey species because virtually all cameras had detections of livestock. For the tiger detection model, we used distance to the nearest roads and distance to nearest water body. We selected these factors based on a literature review and later subjected each to correlation and collinearity analysis. All models successfully converged. We considered a covariate significant if the 95% confidence intervals CI) of its coefficient estimate did not overlap with zero. For this study, we used occupancy as an estimate of space use rather than estimating true occupancy [44,46]. We derived a space use map of tigers based on their space use probability at each of the 94 camera sites from the models. We then used inverse distance weighting (IDW) interpolation in QGIS with the tool ‘IDW Interpolation’ [74] to project tiger space use probability across the study area.

#### (ii) Diel activity and overlap

We calculated diel activity overlap of tigers with their prey species, humans and livestock using the overlap package [69] in program R. We treated three photos per detection as a single detection for diel activity analysis. We used hourly detections of tigers and prey species throughout the diel period to visualize activity overlap. The diel period was first converted to decimal time then multiplied by 2π to convert it to radian time. The overlap value is the common area under the curves using the minimum of the two kernel density estimates for a species pair, ranging from 0 (no overlap) to 1 (complete overlap) [75]. We used 999 bootstraps to generate 95% confidence intervals of overlap values [76]. We used the ‘overlapEst’ function to calculate the estimates of overlap whereas we used the ‘overlapPlot’ function to generate activity overlap plots for each pair of species. We used the function ‘compareCkern’ in the activity package to test the significance of activity overlap between each pair of species.

## 3. Results

### (a) Non-prey covariates

The mean number of human detections was 53.9 ± 297.5, whereas the mean number of livestock detections was 2.9 ± 12.8. Mean per cent canopy cover across the study area was 39.52 ± 16.28 and mean forest cover was 0.712 ± 0.134 km^2^. The mean distance to nearest settlements, roads and permanent water was 3745.6 ± 2110.1 m, 1091.46 ± 1507.6 m and 2785.3 ± 2554.6 m, respectively.

### (b) Spatio-temporal patterns of prey species

**Figure 2.**
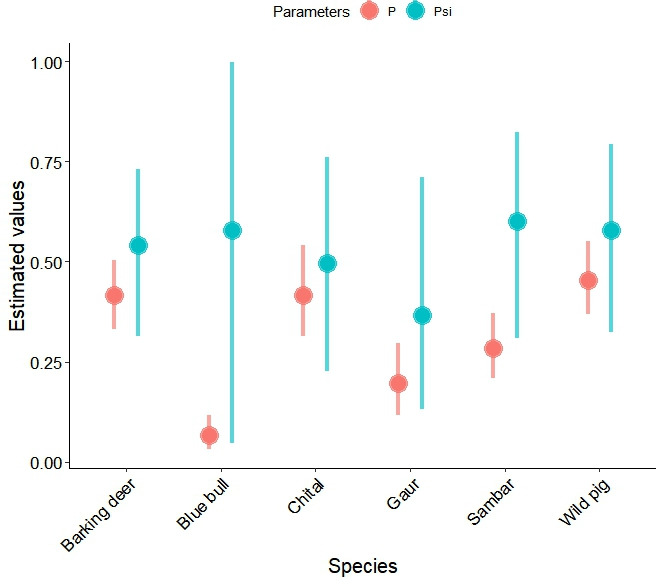
Detection probability (*p*) and space use (*psi*) of major prey species of tigers in PNP and adjoining areas, Nepal. Circles denote mean estimates and error bars denote 99% Bayesian credible intervals.

Among prey species, chital, wild pigs, sambar and barking deer were most commonly detected. We observed the highest detection probability for wild pigs (0.454 ± 0.047), followed by barking deer (0.415 ± 0.044) and chital (0.415 ± 0.058) (table 1; figure 2). The lowest detection probability was observed for blue bull (0.067 ± 0.022). The highest space use was observed for sambar (0.600 ± 0.156), followed by wild pigs (0.579 ± 0.138) and blue bull (0.579 ± 0.327), while the lowest space use was observed for gaur (0.365 ± 0.182). We observed a high activity overlap among prey species but low overlap between prey species with humans and livestock (electronic supplementary material, table S1).

**Table 1.** Detection probability and space use of major prey species of tigers in PNP and adjoining areas, Nepal. (s.d. is the standard deviation; LCL and UCL are the lower and upper 95% confidence limits, respectively; Rhat is the ratio of the variance of a parameter; overlap0 is the Bayesian credible overlap showing the significance of association between tiger space use and covariates (0 represents significance and 1 represents non-significance).)

parameters	mean	s.d.	LCL	UCL	Rhat	overlap
detection probability						
barking deer	0.415	0.044	0.332	0.504	1.000	0
blue bull	0.067	0.022	0.032	0.117	1.000	0
chital	0.415	0.058	0.313	0.542	1.000	0
gaur	0.196	0.046	0.116	0.297	1.000	0
sambar	0.284	0.041	0.210	0.371	1.000	0
wild pig	0.454	0.047	0.368	0.552	1.000	0
space use						
barking deer	0.540	0.125	0.315	0.731	1.000	0
blue bull	0.579	0.329	0.047	0.997	1.000	0
chital	0.497	0.156	0.226	0.760	1.000	0
gaur	0.367	0.183	0.131	0.710	1.000	0
sambar	0.600	0.155	0.308	0.824	1.000	0
wild pig	0.579	0.138	0.324	0.794	1.000	0

### (c) Spatio-temporal patterns of the tigers

**Figure 3.**
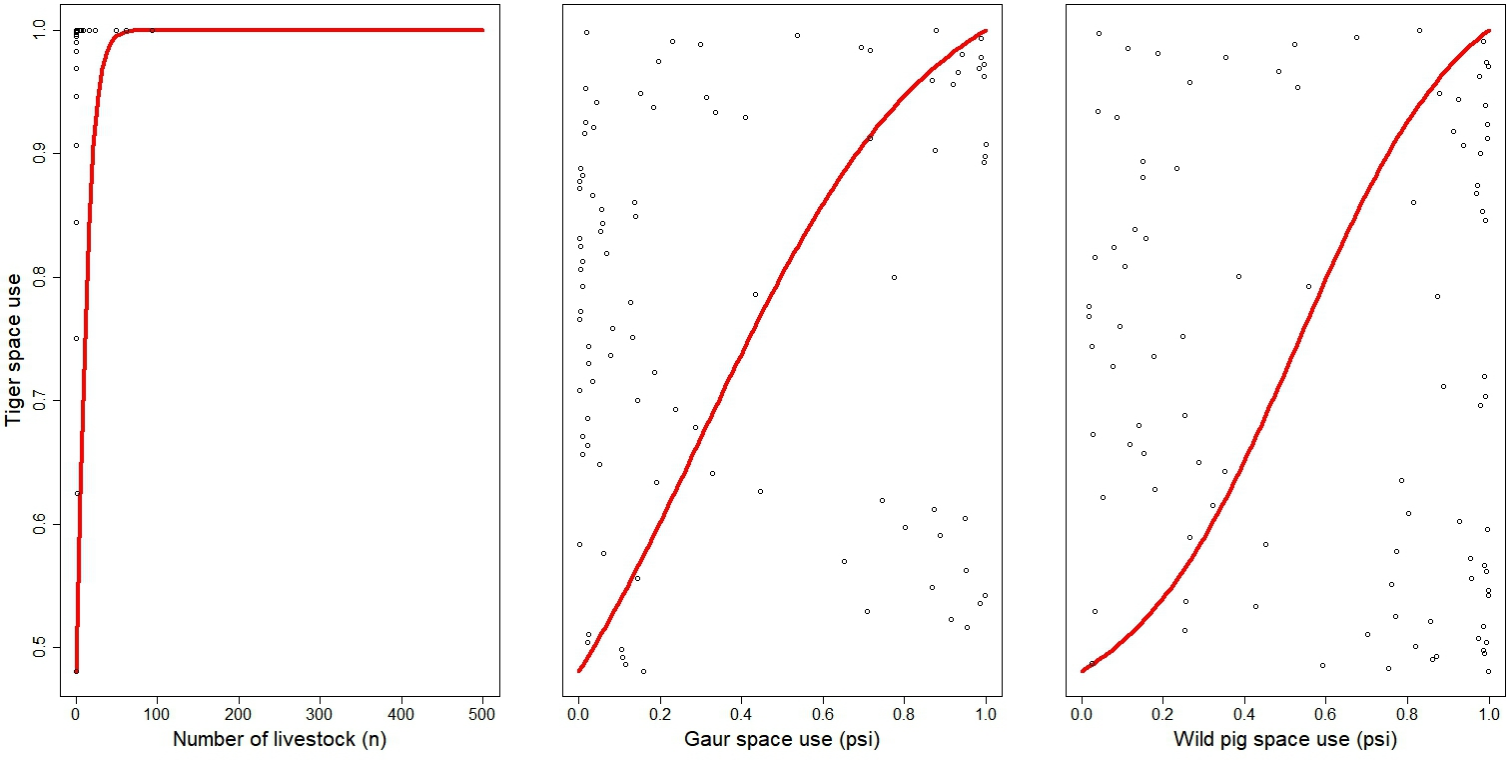
Tiger space use probability in response to number of livestock detections (n), gaur space use probability (psi) and wild pig space use probability (psi) in PNP and adjoining areas, Nepal. White circles represent raw data from tiger space use.

**Figure 4.**
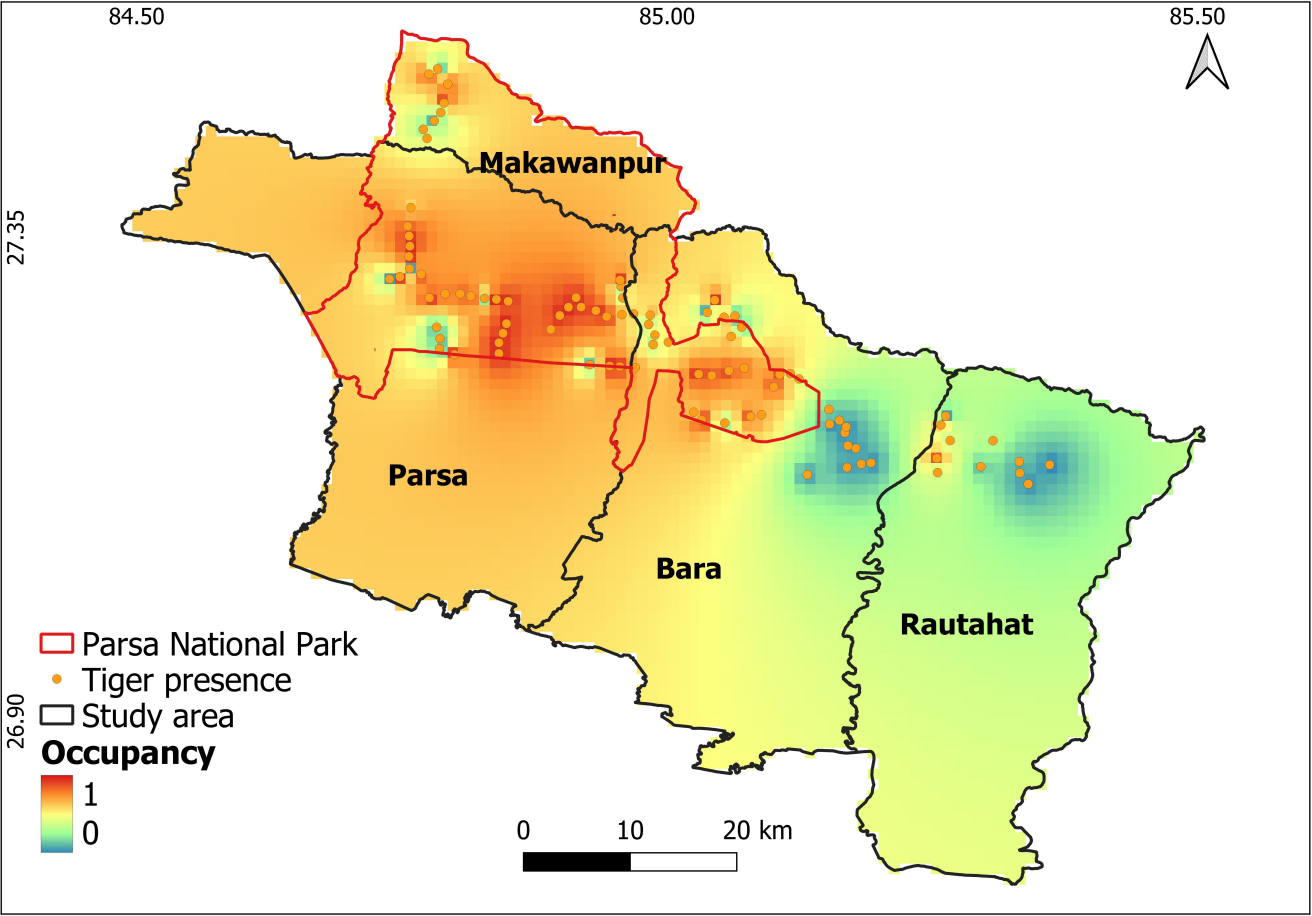
Predicted tiger space use in PNP and adjoining areas, Nepal. Space use was interpolated using probabilities from a hierarchical space use model for tigers.

We detected tigers 201 times across 46 sites. We observed high detection probability (*p* = 0.740 ± 0.073) and moderate space use (psi = 0.543 ± 0.096; table 2; figure 2). Tiger space use was positively associated with that of wild pigs (coefficient: 1.859 ± 0.692), gaur (1.370 ± 0.651) and livestock (1.398 ± 1.228) (figure 3). Tiger detection probability was not influenced by landscape features including distance to road and distance to water (table 2; electronic supplementary material, figure S2). The tiger space use was higher in the western part of the study area, mainly Parsa and Bara districts, whereas their space use in the eastern part was generally low (figure 4).

**Table 2.** Parameter estimates from hierarchical space use models of tigers in PNP and adjoining areas, Nepal. (s.d. is the standard deviation, LCL and UCL are lower and upper 95% confidence limits, respectively; Rhat is the ratio of the variance of a parameter; ESS is the effective sample size; overlap0 is the Bayesian credible overlap showing the significance of association between tiger space use and covariates (0 represents significance and 1 represents non-significance); *p* is the detection probability and psi is the space use of major prey species used in the model.)

parameters	mean	s.d.	LCL	UCL	Rhat	ESS	overlap
*p*	0.740	0.073	0.608	0.895	1.000	13 280	0
psi	0.543	0.096	0.37*	0.718	1.000	78 430	0
space-use model							
*b*0 (intercept)	0.236	0.583	−0.787	1.497	1.000	13 869	1
forest cover (km^2^)	0.648	0.724	−0.625	2.211	1.000	24 951	1
human (*n*)	1.349	1.266	−0.286	4.384	1.000	1 47 000	1
livestock (*n*)	1.398	1.228	0.058	4.604	1.000	1 47 000	0
blue bull (psi)	0.429	0.591	−0.701	1.641	1.000	8790	1
gaur (psi)	1.370	0.561	0.373	2.591	1.000	25 280	0
wild pig (psi)	1.859	0.692	0.698	3.44	1.000	1 47 000	0
farmland area (km^2^)	−2.111	1.814	−4.859	1.756	1.000	8554	1
detection model							
*a*0 (intercept)	2.917	1.129	0.874	4.865	1.000	10 431	0
distance to water (m)	1.070	1.145	−0.726	3.871	1.000	24 088	1
distance to road (m)	1.798	1.364	−0.67	4.589	1.000	34 832	1

**Figure 5.**
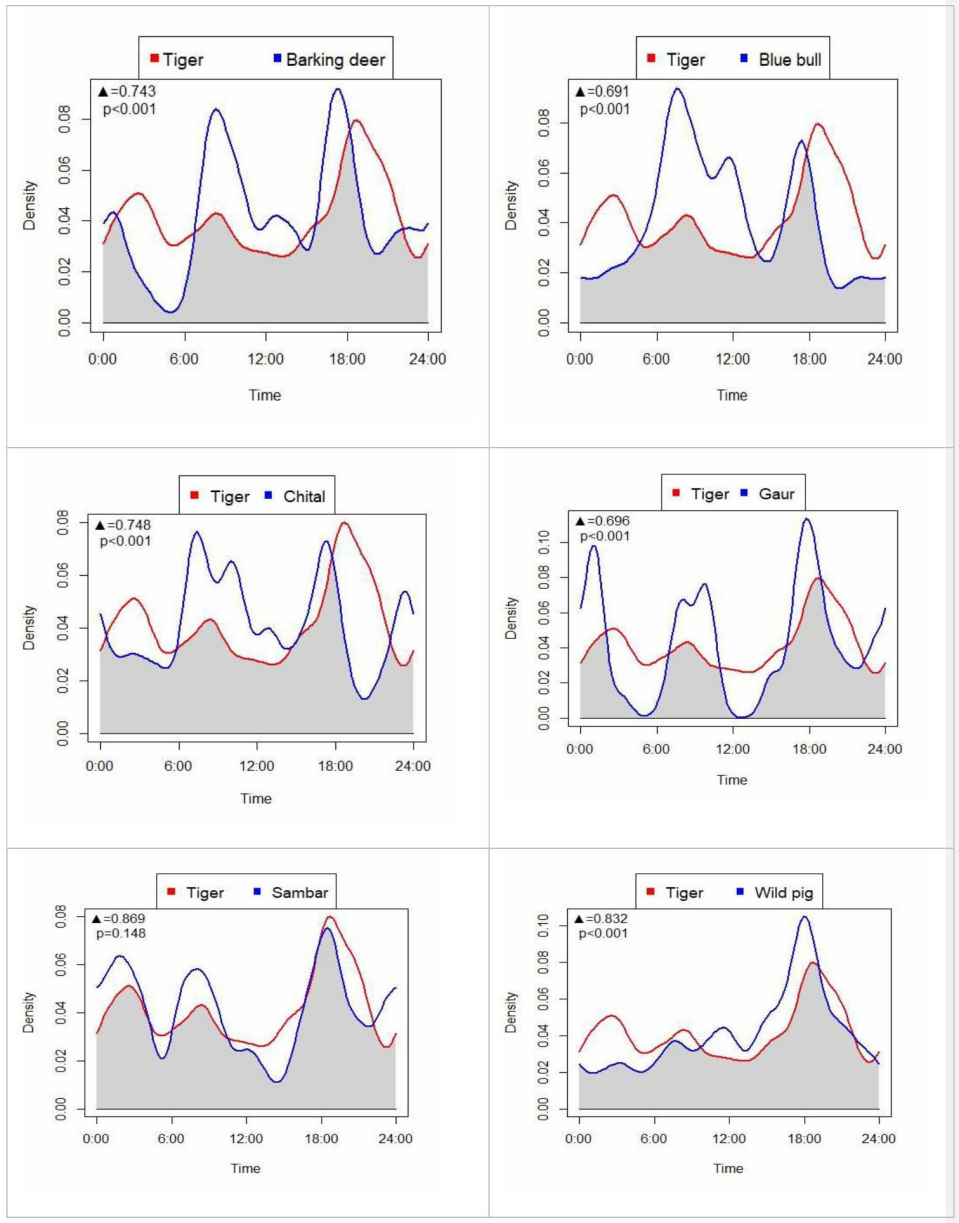
Diel activity overlap between tigers and their major prey species in PNP and adjoining areas, Nepal. Black triangle ▲, denotes the value of activity overlap and *p* denotes significance.

We observed the greatest diel overlap of tigers with sambar (Dhat4 = 0.869, norm0CI = 0.818–0.957), followed by wild pigs (Dhat4 = 0.832, CI = 0.763–0.895) and chital (Dhat = 0.748, CI = 0.649–0.784; figure 5). The least activity overlap of tiger was with blue bull (Dhat = 0.691, CI = 0.599–0.740) and gaur (Dhat = 0.696, CI = 0.567–0.786). Tigers were active throughout the day with the greatest activity during 18.00−20.00 hrs and 2.00−4.00 hrs. Peak activity for sambar was around 18.00 hrs, followed by 2.00−4.00 hrs and 7.00−11.00 hrs, with the former two periods coinciding with peak tiger activity. Wild pigs were active throughout the day with peak activity during 17.00−19.00 hrs which also coincided with peak tiger activity. By contrast, barking deer were most active at 8.00−10.00 hrs and around 18.00 hrs, then activity sharply declined before peak tiger activity.

**Figure 6.**
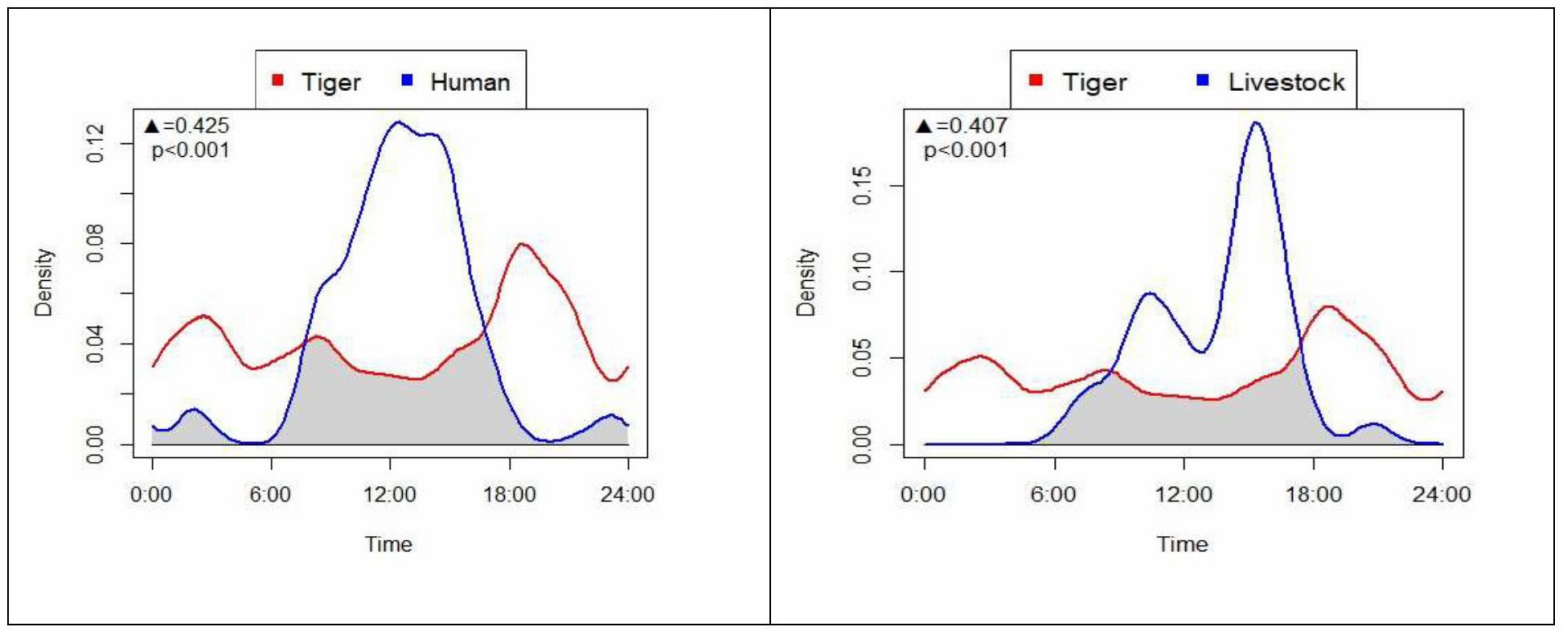
Diel activity overlap of tigers with humans and livestock in PNP and adjoining areas, Nepal. Black triangle ▲, denotes the value of activity overlap and *p* denotes significance.

We observed lower activity overlap between tigers and humans (Dhat4 = 0.425; CI = 0.340–0.464) or livestock (0.407; CI = 0.275–0.445) than that between the tigers and their prey (figure 6). Humans and livestock were diurnal, with greatest human activity during 6.00−18.00 hrs and livestock during 15.00−17.00 hrs.

## 4. Discussion

### (a) Spatio-temporal patterns of the tigers with prey species

Tiger space use indicated that they are widely distributed across the study area, but more concentrated in the northwest region, possibly a consequence of the overall increasing number of tigers in Nepal [25]. PNP, which is in the western part of the study area, contains high-quality tiger habitat [25]. High tiger space use revealed in this study provides evidence for the suitability of PNP and adjoining areas as critical tiger habitat.

Tiger space use and diel activity were largely associated with that of their prey, specifically the wild pigs, gaur and livestock. It was not surprising that wild pigs and gaur, with their large body size and being common in the study area, attract tigers. Although livestock grazing is prohibited inside PNP, people graze their animals near the buffer zone and adjoining areas. For example, 125 livestock depredations were reported in PNP during July 2022–July 2023 [77]. Indeed, wild pigs and gaur have been suggested to be important prey of tigers in the same landscape [8,31] and can comprise a relatively high proportion of biomass in tiger diets [8,57]. These prey species tend to have high space use or detection probability in areas where tigers occur [10,78,79]. Interestingly, we did not find significant associations between tigers and blue bull, despite that they are also considered important prey of tigers [12,15,55]. Owing to collinearity issues, we were unable to independently examine associations between tigers and other prey including chital, barking deer and sambar. The occurrences of chital were positively correlated with that of blue bull (*r* = 0.74), sambar with gaur (*r* = 0.72) and barking deer was positively correlated with gaur (*r* = 0.74) and wild pigs (*r* = 0.75), making it difficult to discern which species are most important to tigers as prey. Nevertheless, each of these species has contributed to the tiger’s prey base in other areas [80], and our study suggests that tigers may prioritize larger prey over smaller ones, though longer-term studies are needed to confirm this.

Similar to observed spatial patterns of the tigers and their prey, tiger diel activity extensively overlapped with that of wild pigs, further suggesting their importance as prey [30]. The highly synchronized temporal activities between tigers and wild pigs in the study area indicates that the tigers probably hunt during the evenings and mornings when chances of encountering this prey are high, which has implications in how we may avoid human–tiger encounters. However, high observed overlap does not necessarily imply that large predators are killing the preferred prey species [80], as space use by predators can be more strongly associated with prey species abundance. For example, abundance of chital and sambar influenced space use of tiger in Chitwan National Park [81]. Despite varied responses of tigers to different prey species revealed in this study, we caution that our single-season data may miss seasonal effects of prey fluctuations on tigers, which warrant multi-season studies of prey selection.

### (b) Spatio-temporal patterns of the tigers in relation to anthropogenic activities

Our expectation that the tigers would spatially avoid livestock and humans was not supported, in contrast with previous studies. For instance, livestock presence has been negatively associated with detectability of tigers [81]. Similarly, other studies [10,19] suggested that tigers select for areas with lower human disturbances. Our findings that tigers did not spatially avoid humans, and spatially overlapped with livestock, highlights potential risks of human encounters and livestock depredation, which may escalate human–tiger conflicts. Interestingly, there was little overlap in diel activity between tigers and humans or livestock, suggesting temporal avoidance. Humans and livestock were primarily diurnal, with peak activity around 15.00−17.00. Given the perceived threat of encountering large predators, humans and livestock might have adjusted their diel activity [82,83]. Tigers, by contrast, are also reported to avoid human-disturbed areas [9,10] and may use temporal avoidance to reduce human encounters, as observed in other areas of Nepal, including Chitwan National Park [9,19].

Human–tiger conflicts could be potentially reduced through temporal segregation strategies, increasing the resilience and adaptability of humans and livestock in the presence of large predators [9,10,56,84]. Furthermore, the presence of a highway crossing through the park may create spatial and temporal segregation, as the movements of humans and livestock would be somewhat constrained by the road. Therefore, by maintaining spatial segregation between tigers and humans/livestock, human–tiger conflicts could be managed in this system. However, many factors, such as local cultural practices, farming and grazing practices, landscape features and tiger behavioural flexibility, will need to be carefully considered if relying on mutual temporal avoidance to maintain human–tiger coexistence. The complex nature of human–wildlife interactions requires detailed and localized knowledge about spatial and temporal patterns of all species involved [85].

#### (c) Conservation implications for tigers in Parsa National Park and adjoining areas

The PNP contains critical habitat for tigers, currently supporting 41 individuals [25]. However, considering that PNP has a limited area (627.39 km^2^ for PNP with a buffer zone of 285.30 km^2^ [25]), the tiger population could experience intraspecific competition for space, prey, mates [86,87] and sympatric predator species [41,80]. Our results suggest that space use of prey species was high across PNP and adjoining forests, suggesting prey availability may be similar between PNP and adjoining forests. Though pervasive human use negatively impacted tiger occupancy in Chitwan National Park [81], tiger space use and number of human detections were positively associated in PNP. This could be owing to national or community forests having high human activities such as timber collection, livestock grazing, fodder collection and illegal hunting [76,77]. Therefore, they represent areas of high probability of human–tiger interactions. Restricting human activities spatially or temporally, conducting community education programmes on predator behaviour, improving livestock enclosures, and adopting practices like rotational grazing which have potential to reduce human and livestock losses, could facilitate human–tiger coexistence in these forests.

## 5. Conclusions

Our findings, though of limited duration, provide valuable insights into the spatio-temporal patterns of tigers in PNP and adjoining areas of Nepal. Considering that tigers are globally endangered, and local information is critical for each small population, this study offers timely information to help guide conservation strategies. Our study confirms that tigers are widely distributed across the study area, appear to closely track their prey in space and time, and may use temporal avoidance to achieve spatial co-occurrence with humans. Furthermore, we identified that forests outside the PNP, such as forests in Bara and Rautahat districts, contain suitable habitats for tigers, whereas the southern area is less suitable owing to the presence of large human settlements and low prey availability. These adjoining forests with suitable prey availability not only can provide more space and resources for tigers, but offer opportunities for designing and testing the effectiveness of human–tiger conflict mitigation strategies. The PNP, along with Chitwan National Park, play a key role in securing the future of human–tiger coexistence in the TAL. Following the recent increase in tiger populations in Nepal, the next step is to improve human–tiger coexistence. Managing tiger habitats inside protected areas will not be sufficient and understanding tiger behaviour in human-dominated landscapes is key to their long-term conservation. For this, long-term studies, especially multi-year studies, remain urgently needed to ensure that conservation practices remain relevant to the spatio-temporal dynamics of tigers.

## Data Availability

Our camera trap data are from fixed locations where we detected endangered species including tigers. Because of the potential for poachers to use these sites to locate target species, we cannot make our presence data publicly available. However, we can provide these data on reasonable request to the corresponding author. The data except presence location of species are deposited at Dryad [88]. Electronic supplementary material is available online [89].
